# Targeting glucosylceramide synthase upregulation reverts sorafenib resistance in experimental hepatocellular carcinoma

**DOI:** 10.18632/oncotarget.6982

**Published:** 2016-01-22

**Authors:** Milica Stefanovic, Anna Tutusaus, Guillermo A. Martinez-Nieto, Cristina Bárcena, Estefania de Gregorio, Catia Moutinho, Elisabet Barbero-Camps, Alberto Villanueva, Anna Colell, Montserrat Marí, Carmen García-Ruiz, Jose C. Fernandez-Checa, Albert Morales

**Affiliations:** ^1^ Department of Cell Death and Proliferation, IIBB-CSIC, IDIBAPS, Barcelona, Catalonia, Spain; ^2^ Cancer Epigenetics and Biology Program (PEBC), Bellvitge Biomedical Research Institute, Barcelona, Catalonia, Spain; ^3^ Translational Research Laboratory, Catalan Institute of Oncology - Bellvitge Biomedical Research Institute, Barcelona, Catalonia, Spain; ^4^ Liver Unit, Hospital Clinic, CIBEREHD, Barcelona, Catalonia, Spain; ^5^ Research Center for Alcoholic Liver and Pancreatic Diseases, Keck School of Medicine of the University of Southern California, Los Angeles, CA, USA

**Keywords:** liver cancer, chemotherapy, mitochondria, ceramide, mouse model

## Abstract

Evasive mechanisms triggered by the tyrosine kinase inhibitor sorafenib reduce its efficacy in hepatocellular carcinoma (HCC) treatment. Drug-resistant cancer cells frequently exhibit sphingolipid dysregulation, reducing chemotherapeutic cytotoxicity via the induction of ceramide-degrading enzymes. However, the role of ceramide in sorafenib therapy and resistance in HCC has not been clearly established. Our data reveals that ceramide-modifying enzymes, particularly glucosylceramide synthase (GCS), are upregulated during sorafenib treatment in hepatoma cells (HepG2 and Hep3B), and more importantly, in sorafenib-resistant cell lines. GCS silencing or pharmacological GCS inhibition sensitized hepatoma cells to sorafenib exposure. GCS inhibition, combined with sorafenib, triggered cytochrome c release and ATP depletion in sorafenib-treated hepatoma cells, leading to mitochondrial cell death after energetic collapse. Conversely, genetic GCS overexpression increased sorafenib resistance. Of interest, GCS inhibition improved sorafenib effectiveness in a xenograft mouse model, recovering drug sensitivity of sorafenib-resistant tumors in mice. In conclusion, our results reveal GCS induction as a mechanism of sorafenib resistance, suggesting that GCS targeting may be a novel strategy to increase sorafenib efficacy in HCC management, and point to target the mitochondria as the subcellular location where sorafenib therapy could be potentiated.

## INTRODUCTION

Hepatocellular carcinoma (HCC) is the most common liver cancer and the end stage of chronic liver disease [1]. Its prevalence is expected to rise due to the escalating increase of non-alcoholic fatty liver disease associated to obesity and metabolic syndrome, and the incidence of HCV [2, 3]. HCC is often diagnosed in an advanced stage characterized by resistance to current therapy, when curative strategies are no longer applicable. The establishment of the multikinase inhibitor sorafenib as the standard of care has opened a window of hope for HCC patients with very poor prognosis [3]. However, this promising systemic treatment has limited survival benefits with low rates of tumor response, probably due to the existence of primary and acquired drug resistance mechanisms [4, 5]. Several drugs are now in the pipeline for HCC application, either alone or in combination with sorafenib, but the lack of positive results complicates their clinical application. Additionally, effective therapy combinations may reveal novel targets of treatment for HCC [4–6].

Ceramide is a bioactive sphingolipid generated in response to a wide range of stimuli, including chemotherapeutic agents, which triggers cell death [7]. Transient or sustained ceramide generation (Suppl. Fig. 1), either by sphingomyelinases activation or *de novo* synthesis, respectively [7, 8], can be limited by the concurrent activation of ceramide-degrading enzymes, which reduce the efficacy of drug therapy on tumor cells [8, 9]. For instance, glucosylceramide synthase (GCS) catalyzes the generation of glucosylceramide from ceramide while ceramidases (CDases) deacylate ceramide to sphingosine, which is then phosphorylated to sphingosine-1-phosphate by sphingosine kinases. Both pathways have been characterized in drug-resistance as protective mechanisms triggered by tumor cells after cancer treatment [8, 10, 11]. In liver cancer, increasing intratumoral ceramide levels with nanoliposomal administration has been used as a strategy in the treatment of HCC [12], while targeting acid CDase (ACDase) potentiated the cytotoxic effect of daunorubicin in hepatoma cells [13]. Regarding sorafenib action, recent data has shown the efficacy of combining sorafenib with recombinant acid sphingomyelinase, a ceramide-generating enzyme, in experimental liver cancer [14], or with nanoliposomal ceramide in melanoma or breast cancer [15]. These findings have proposed a role for sphingolipids in sorafenib toxicity [16], but a detailed analysis of ceramide metabolism *in vitro* and *in vivo* HCC models after sorafenib treatment has not been previously reported.

Our data indicate that, although sorafenib alters the sphingolipidic metabolism in hepatoma cells via ASMase activation, ceramide toxicity is partially reduced by the simultaneous induction of ceramide-eliminating enzymes, in particular GCS. Moreover, pharmacological or genetic GCS antagonism sensitized hepatoma cells to sorafenib by a caspase-independent mitochondrial-dependent mechanism. Moreover, GCS is upregulated in resistant hepatoma cells after long-term exposure to sorafenib, pointing to GCS targeting as an effective approach to re-sensitize tumor cells to sorafenib. Therefore, our results validate the interest of ceramide-focused strategies to increase sorafenib effectiveness in HCC and confirm mitochondria as the subcellular site responsible for these effects.

## RESULTS

### Sorafenib increases ceramide levels and the expression of enzymes involved in ceramide metabolism in Hep3B cells

Despite several evidences showing the influence of ceramide-related compounds in sorafenib efficacy [14, 15], the effect of sorafenib on ceramide metabolism has not been evaluated. Among critical sphingolipidic genes (Suppl. Fig. 1), we found that overnight sorafenib exposure increased expression of genes responsible for ceramide production (Table 1) by sphingomyelin hydrolysis (acid sphingomyelinase, ASMase) or *de novo* synthesis (serine palmitoyl transferase, SPT, ceramide synthase 2, CerS2). In parallel, genes involved in ceramide modification via ceramidase degradation (acid ceramidase, ACDase, and sphingosine kinase 1, SK1) or glycosylation (glucosylceramide synthase, GCS) were also increased. Moreover, in another hepatoma cell line, HepG2, sorafenib also increased ceramide formation through ASMase and glycosylation via GCS (Suppl. Table 1).

**Table 1:** mRNA levels of main sphingolipidic enzymes in Hep3B cells after sorafenib exposure

Sorafenib (μM)	0	2.5	5	10
ASMase	1.00±0.32	2.05±0.66 *	2.39±0.22 *	1.53±0.60
NSMase	1.00±0.10	0.90±0.20	1.10±0.20	1.15±0.15
ACDase	1.00±0.07	2.07±0.59 *	2.70±0.37 *	1.33±0.56
NCDase	1.00±0.11	0.85±0.16	0.95±0.23	1.05±0.20
CerS2	1.00±0.35	1.63±0.54	1.85±0.70	2.65±0.69 *
CerS4	1.00±0.21	1.10±0.09	1.20±0.29	1.30±0.18
GCS	1.00±0.09	1.65±0.73	2.46±0.34 *	4.01±0.69 *
SPT	1.00±0.13	1.59±0.54	2.10±0.33 *	1.97±0.59 *
SK1	1.00±0.37	1.40±0.35	1.64±0.45	0.77±0.51

Hep3B cells were exposed to increasing doses of sorafenib (2.5, 5, 10 μM) for 16 hours and main enzymes in ceramide metabolism analyzed by RT-PCR. (n=3).

* p<0.05 vs. control.

**Figure 1:**
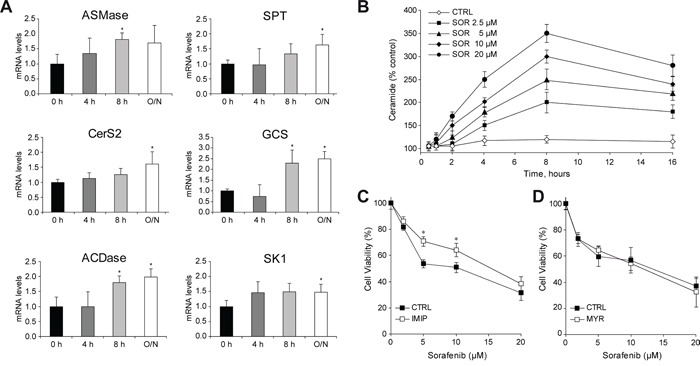
Sorafenib administration to hepatoma cells induces changes in ceramide metabolism **A.** Time-course analysis of mRNA levels of main sphingolipidic enzymes in Hep3B cells exposed to sorafenib (5μM). **B.** Ceramide levels were quantified in Hep3B cells treated with increased doses of SOR (2.5, 5, 10 and 20 μM) during different times of incubation, after lipid extraction, TLC running and PhosphoImager quantification. **C.** and **D.** Cell viability of Hep3B cells, preincubated (30 min) with imipramine (IMIP, 15 μM) or myriocin (MYR, 5 μM), and treated with sorafenib for 16 h. (n=3). *, p<0.05 vs. control.

Rapid changes in ceramide concentration due to ionizing radiation or chemotherapeutic agents are induced by ASMase stimulation, while sustained ceramide increase via de novo synthesis occurs through activation of ceramide synthases, such as CerS2 and CerS4, which exhibit predominant liver expression [20, 21]. Time-response analysis in Hep3B cells showed both increases (Figure 1A), in ASMase and in *de novo* ceramide synthesis (SPT and CerS2). Moreover, sorafenib induced the expression of GCS and ACDase, which metabolize ceramide, as well as SK1. These effects were accompanied by changes in ceramide levels upon sorafenib treatment. Ceramide increased dose-dependently, being significant for all doses (from 2.5 to 20 μM) after 4 h of sorafenib exposure (Figure 1B).

### Pharmacologic inhibition of sphingolipid enzymes modulates sorafenib-induced toxicity in hepatoma cells

To examine the role of the ceramide production/degradation pathways in sorafenib cytotoxicity, we administered sphingolipid inhibitors combined with sorafenib in hepatoma cells (Suppl. Fig. 1). First, we used myriocin (MYR, 5 μM), which targets ceramide *de novo* biosynthesis by inhibiting SPT; and imipramine (IMIP, 15 μM), tricyclic antidepressant and effective ASMase inhibitor [22], to block ceramide generation from the sphingomyelin pathway, at doses that caused no effect in hepatoma cell growing. Imipramine reduced significantly sorafenib-induced cell death (Figure 1C), while myriocin (Figure 1D) or fumonisin B1 (FB1) (data not shown), another blocker of the *de novo* pathway [22], caused no effect in sorafenib action, further confirming a contributory role of ASMase activation in sorafenib toxicity [14].

**Figure 2:**
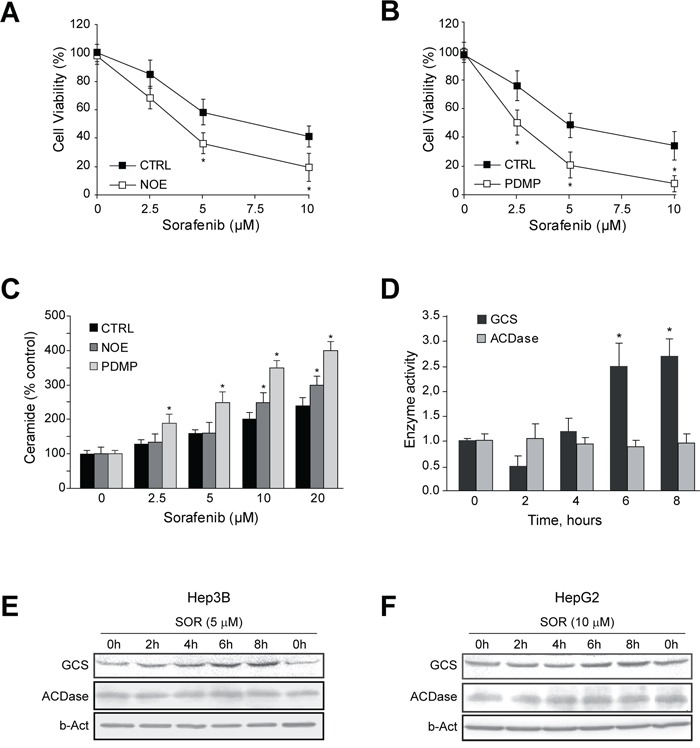
GCS is activated by sorafenib and GCS inhibition increases sorafenib toxicity in hepatoma cells **A.** and **B.** Cell viability of Hep3B cells, preincubated (30 min) with NOE (100 μM) or PDMP (30 μM), and treated with sorafenib for 16 h. **C.** Ceramide levels were quantified in Hep3B cells, preincubated with NOE and PDMP, and sorafenib for 4h. **D.** Time-course analysis of GCS and ACDase activities were analyzed in Hep3B cells treated with sorafenib (10 μM). **E.** and **F.** Hep3B and HepG2 cells, respectively, were treated with sorafenib and GCS protein levels measured at different times. *, p<0.05 vs. control cells. RNA interference was validated by qPCR and protein levels of GCS (E) and ACDase (F). Cell viability after sorafenib treatment was measured in GCS- and ACDase-silenced Hep3B cells, respectively, and compared to siCTRL-cells.

To examine if forcing ceramide accumulation could increase sorafenib cytotoxicity *in vitro*, we tested cell viability after inhibition of ACDase with NOE (Figure 2A), or GCS with PDMP in sorafenib-treated Hep3B cells (Figure 2B). Cell death was augmented significantly upon inhibition of GCS and ACDase, and similar effects were observed with PDMP but not NOE in sorafenib-exposed HepG2 cells (Suppl. Fig. 2). Of note, neither PDMP nor NOE alone caused damage to primary mouse hepatocytes as previously reported [13, 18], having no significant effect on sorafenib toxicity in normal hepatocytes (data not shown). Moreover, changes in ceramide content after inhibition of ACDase with NOE or GCS with PDMP were confirmed in sorafenib-treated Hep3B cells (Figure 2C). Remarkably, GCS inhibition was more effective increasing ceramide levels after sorafenib exposure, in line with greater sorafenib toxicity induced by PDMP, and further demonstrating the upregulation of ceramide metabolism after sorafenib exposure. Interestingly, we detected GCS but not in ACDase induction by sorafenib (Figure 2D) in Hep3B cells, despite of increased ACDase (Figure 1A). These results were confirmed by western blot in samples from Hep3B (Figure 2E) and in HepG2 cells (Figure 2F), paralleling the increase seen at the mRNA levels of GCS in both hepatoma cell lines after sorafenib addition. Therefore, our results indicated that the blockage of ceramide-modifying enzymes, particularly GCS, potentiates ceramide contribution to sorafenib toxicity.

### GCS silencing potentiates sorafenib-induced toxicity in hepatoma cells

**Figure 3:**
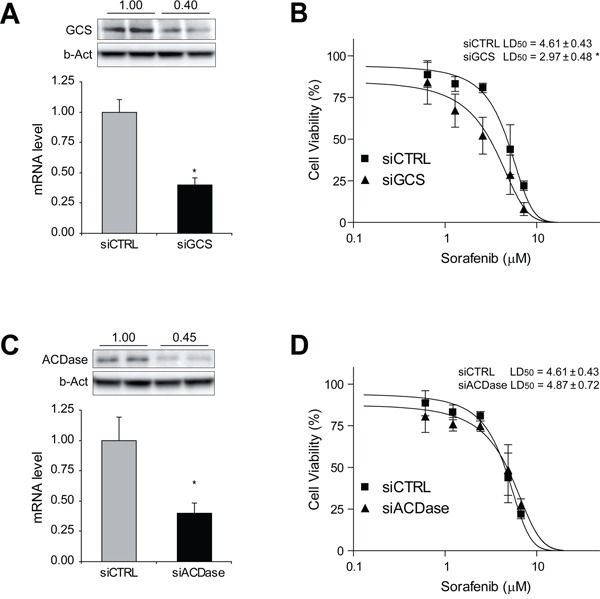
GCS silencing sensitizes hepatoma cells against sorafenib exposure Hep3B cells were transfected with siRNA control and against GCS and ACDase. RNA interference was validated by qPCR and protein levels of GCS **A.** and ACDase **C.** Cell viability after sorafenib treatment was measured in GCS- and ACDase-silenced Hep3B cells (**B** and **D**, respectively) and compared to siCTRL cells. *, p<0.05 vs. siCTRL cells.

To further verify the contribution of ceramide in sorafenib cytotoxicity, hepatoma cells were transfected with siRNAs against GCS and ACDase analyzing sorafenib-induced cell death. GCS silencing in Hep3B cells, as detected by mRNA and protein levels (Figure 3A), elicited increased sorafenib toxicity (Figure 3B). Similarly, HepG2 cells transfected with GCS siRNA displayed higher sensitivity to sorafenib (Suppl. Fig. 3). However, ACDase silencing (Figure 3C), did not sensitize Hep3B cells sorafenib (Figure 3D), in discrepancy with the results observed after NOE inhibition, maybe suggesting NOE off-target effects. Moreover, silencing GCS in PLC cells, another hepatoma cell line, failed to sensitize to sorafenib toxicity (Suppl. Fig. 4), and this effect was accompanied by a modest reduction of GCS protein levels (30-40%). However, pharmacological inhibition of GCS with PDMP was highly effective in sensitizing PLC cells to sorafenib (Suppl. Fig. 5), although it required higher doses of PDMP compared to other hepatoma cell lines. Overall, our data suggest that blocking ceramide elimination via GCS reduction, rather than ACDase, improve sorafenib cytotoxicity in HCC cells, clearly pointing to GCS as the sphingolipidic enzyme to pharmacologically target for sorafenib combined therapy.

### GCS inhibition reduced tumor growth in subcutaneous HCC mouse model after sorafenib treatment

**Figure 4:**
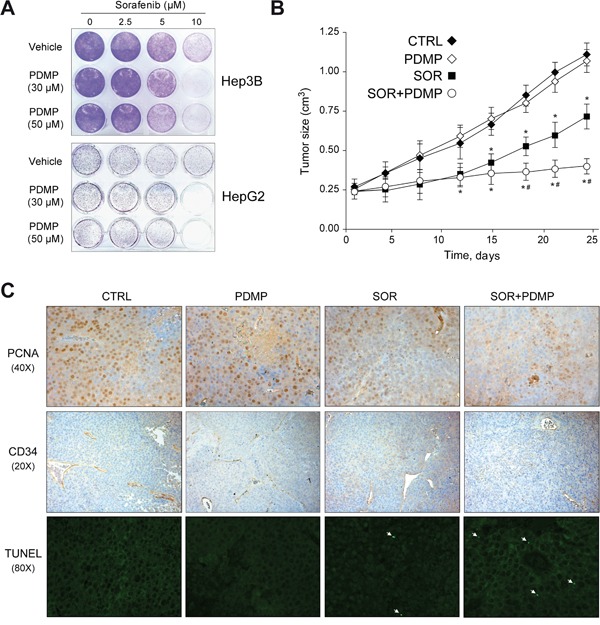
GCS inhibition reduces subcutaneous tumor growth in mouse **A.** Hep3B (upper) and HepG2 (lower) cells were treated with PDMP (0, 30 and 50 μM) and sorafenib (0, 2.5, 5 and 10 μM) for 1 day, culture medium changed, and cells allowed to grow for three extra days. Crystal violet staining was performed to illustrate changes in colony formation and representative images taken. **B**. Mice bearing HepG2-subcutaneous tumors were i.p. injected with PDMP (60 mg/kg) and sorafenib given orally by gavage (80 mg/kg) daily for 3 weeks (CTRL, n=8; PDMP, n=6; SOR+PDMP, n=8). *, p<0.05 vs. vehicle-treated mice. #, p<0.05 vs. sorafenib-treated mice. **C.** Representative images of tumor samples stained for PCNA, CD34 and TUNEL detection.

Before starting *in vivo* treatments, in order to replicate the findings of sensitization to sorafenib upon GCS inhibition/silencing seen with the MTT-based cell viability approach, crystal violet cell proliferation assay was used to measure the number of viable cells. After four days exposure, Hep3B and HepG2 cells treated with PDMP were clearly sensitized to sorafenib, diminishing cell survival after GCS inhibition (Figure 4A), reproducing in clonogenic assays the findings observed with the MTT assay. After that, we established bilateral xenograft tumors by injecting subcutaneously HepG2 cells in the flanks of nude mice. Once measurable tumors were established, animals received sorafenib or vehicle by oral gavage, with or without PDMP i.p administration. While sorafenib-treated mice exhibited reduced tumor growth, this effect was potentiated by PDMP treatment (Figure 4B). In addition, the sensitizing effect of GCS inhibition by PDMP was accompanied by reduced tumor cell proliferation, as denoted by PCNA detection (Figure 4C) and vascularization, as detected in CD34 stained slides (Figure 4C). Moreover, we performed TUNEL assay in our samples to identify DNA fragmentation as consequence of the apoptotic cell death induced by the chemotherapeutic treatments (Figure 4C). Only few TUNEL positive cells were identified after sorafenib treatment, number that was slightly increased after PDMP co-treatment. In fact, the percentage of cells detected with fragmented nuclear DNA is low (under 1%), maybe suggesting that PDMP/sorafenib combination is not inducing classical apoptotic cell death. Of note, PDMP alone administration in mice did not modify tumor growth (Figure 4B), vessel formation (Figure 4C) or induce any hepatic damage to treated animals (data not shown). Therefore, GCS pharmacological inhibition was effective in increasing the efficacy of sorafenib therapy in mice bearing subcutaneous hepatoma tumors.

### Effect of GCS inhibition on sorafenib-induced anti-proliferative effects and autophagy in hepatoma cells

**Figure 5:**
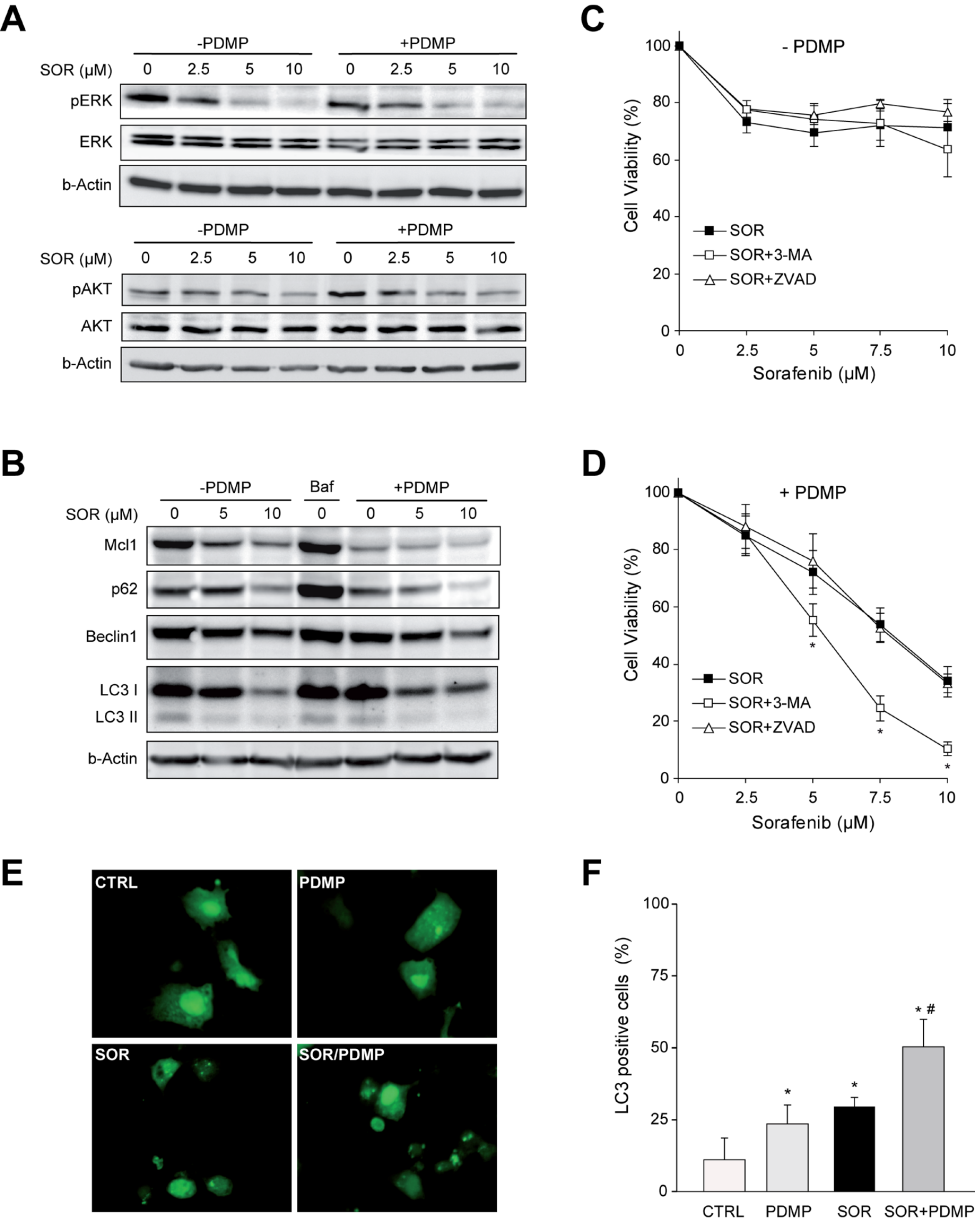
Signaling changes after GCS inhibition in sorafenib treated hepatoma cells **A.** Protein levels of ERK and AKT phosphorylation in Hep3B cells after 16 h exposure to increasing doses of sorafenib (2.5, 5, 10 μM) with or without PDMP (30 μM, 4 h) for 4 h. **B.** Expression levels of Mcl1, Beclin 1, p62 and LC3 were analyzed by Western blot and β-actin used as a loading control. Bafilomycin A1 (Baf, 0.1 μM) was used as autophagy inhibitor. **C.** and **D.** Cell viability of Hep3B cells pretreated (30 min) with autophagy inhibitor (3MA, 2 μM) or pancaspase inhibitor Z-VAD-FMK (ZVAD, 25 μM) before sorafenib/PDMP exposure for 16 hours. *, p<0.05 vs. control Hep3B cells. **E.** Hep3B cells expressing GFP-LC3 were treated with sorafenib and/or PDMP and representative images were taken 6 hours later. **F.** Wide-field pictures were taken and the number of GFP-LC3-positive autophagosomes per cell was counted in 100 cells per condition. Hep3B cells exhibiting three or more green puncta per cell were considered positive. *, p<0.05 vs. control Hep3B cells. ^#^, p<0.05 vs. sorafenib Hep3B cells.

We next explored potential mechanisms underlying the potentiation of SOR-induced cell death by GCS inhibition. Since the RAF/MEK/ERK pathway and PI3K/AKT activity are critical in the progression of HCC [23], we analyzed the impact of GCS inhibition with PDMP on these pathways. Following sorafenib exposure, pAKT and pERK decreased in a dose-dependent manner in Hep3B cells (Figure 5A). However, no additional changes on PI3K/AKT and RAF/MAPK/ERK signaling pathway were detected after GCS inhibition.

Ceramide accumulation [24] and sorafenib exposure [25] have been reported to promote autophagy. To determine if enhanced cytotoxic effect of combined therapy was associated with autophagic cell death, we treated hepatoma cell line Hep3B with sorafenib and tracked the conversion of LC3-I to the LC3-II form, as an autophagosome marker, and p62, which is ubiquitinated and degraded by the autolysosomes. Sorafenib induced a reduction in LC3-I that was accompanied by enhanced degradation of p62, consistent with an increase in autophagy after the treatment (Figure 5B). LC3-II accumulation was not evident, probably because LC3-II is also degraded by autophagy. The sorafenib/PDMP treatment resulted in decreased p62 levels compared to sorafenib alone suggesting that ceramide accumulation by PDMP potentiates sorafenib-induced autophagy, although not providing conclusive data. To further analyze this event, GFP-LC3 expressing Hep3B cells were cultured in the presence or absence of sorafenib and/or PDMP and the levels of punctate LC3-positive autophagosomes in cells were calculated in each condition [26]. As seen, sorafenib plus PDMP increased the number of LC3 positive dots, indicating enhanced autophagosome formation (Figure 5E) as denoted by the quantification of the percentage of cells with three or more autophagosomal puncta (Figure 5F).

Beclin 1 is a Bcl-2-homology domain 3 (BH3)-only protein required for the formation of the autophagosome, and Mcl1 is an anti-apoptotic Bcl-2 homolog that inhibits autophagy by “sequestering” Beclin 1 in a dimer [27]. Both proteins have a vital role in autophagy regulation in HCC cells [28]. Our data showed that sorafenib markedly decrease Mcl1 levels, and this effect is potentiated by PDMP (Figure 5B). In fact, Beclin 1/Mcl1 ratio increased from 1.0 in control cells to 2.8 in PDMP-sorafenib-treated cells, probably releasing Beclin 1 molecules to promote autophagy. In addition, we have tested for Bcl-2 and Bcl-xL levels after sorafenib treatment and have found no differences, at least at short-time incubation, which contrasts with the sharp decline observed for Mcl-1 levels as soon as 2-4 hours of sorafenib exposure (data not shown).

Autophagy has a dual role in cancer cells either promoting survival by providing nutrients to proliferating cells or triggering cell death via lethal mitophagy [26]. Moreover, GCS inhibitors have been recently described as enhancers of autophagy flux in primary neurons [29]. To address whether sorafenib plus PDMP-induced autophagy is protective or toxic, we incubated sorafenib/PDMP-treated cells with 3-methyladenine (3-MA), inhibitor of autophagy initiation. We first tested different 3-MA concentrations to guarantee autophagy inhibition in the absence of cytotoxicity to hepatoma cells. After that, 3-MA-induced autophagy inhibition displayed small protection against sorafenib alone (Figure 5C), while cell death was clearly potentiated by 3-MA in the combined treatment (Figure 5D). Therefore, sorafenib/PDMP-induced autophagy induction seems to act as a protective mechanism, discarding autophagy-induced cell death as the mechanism triggered during PDMP/sorafenib toxicity.

### GCS inhibition triggers mitochondrial-dependent cell death by sorafenib in hepatoma cells

**Figure 6:**
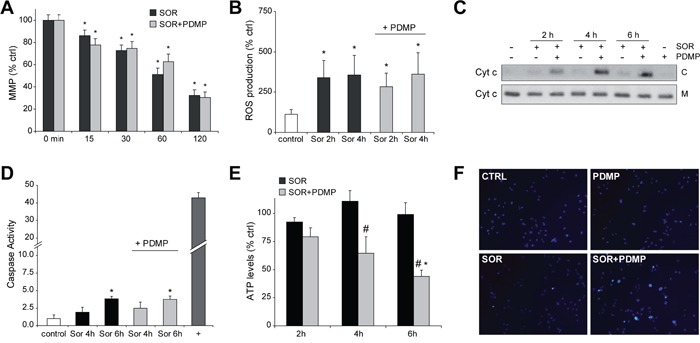
GCS inhibition induces cytochrome c release and ATP depletion to sorafenib-treated hepatoma cells **A.** Mitochondrial membrane potentialby JC1determination and **B.** ROS production by DCF quantification was determined in Hep3B cells exposed to sorafenib (10 μM) and PDMP (50 μM). **C.** cytochrome c levels in cytosol (C) and mitochondria (M) were analyzed by western blot in cell extracts from sorafenib/PDMP treated cells. **D.** Fold increase in caspase 3 activity was determined in total cell extracts as above, and TNF (50 ng/ml) plus cycloheximide (40 μM) used as a positive control (+). **E.** and **F.** ATP levels were measured in Hep3B cells treated with sorafenib and compared to sorafenib+PDMP combination. *, p<0.05 vs. control Hep3B cells. #, p<0.05 vs. sorafenib-treated Hep3B cells. **F.** Nuclear Hoechst staining was visualized in Hep3B cells treated with sorafenib and/or PDMP.

Besides its involvement in autophagy regulation, Mcl1 is an antiapoptotic mitochondrial Bcl-2 member, suggesting that the sensitization of PDMP to sorafenib could involve apoptotic cell death. Mitochondrial damage results from sorafenib interaction with mitochondrial respiratory chain and reactive oxygen species (ROS) production [30]. Similarly, ceramide induces mitochondrial permeability after direct interaction with complex III of the respiratory system [31]. Moreover, sphingolipids, and particularly ceramide, promote changes in mitochondrial membrane composition favoring channel formation by Bcl-2 family members [32, 33]. Therefore, we evaluated the influence of GCS inhibition on ROS production and mitochondrial membrane potential (MMP) after sorafenib treatment. Hep3B cells were treated with sorafenib and/or PDMP and incubated for 30 minutes with DCF to determine ROS production and with JC-1 to estimate MMP. Sorafenib induced a rapid decline in MMP even at low doses, which was not modified by PDMP addition (Figure 6A). In fact, dissipation of MMP was complete in less than 30 minutes with sorafenib doses over 10 μM (not shown here), while PDMP alone had no affect. In parallel, ROS induction caused by sorafenib was not potentiated by PDMP, as measured fluorimetrically by DCF (Figure 6B). In line with these observations, we addressed whether sorafenib-induced mitochondrial complex I inactivation, as observed in human neuroblastoma cells [30] is potentiated by sorafenib. Complex I activity decreased in sorafenib-treated hepatoma cells (around 50%), but PDMP co-addition did not significantly modify it (Suppl. Fig. 6).

Interestingly, despite the lack of changes in ROS and MMP by GCS inhibition in sorafenib-treated HCC cells, combined drug treatment enhanced the release of cytochrome c into the cytosol (Figure 6C), indicative of mitochondrial outer membrane permeabilization. Of note, neither sorafenib (1-10 μM) nor PDMP alone up to 6-8 hours exposure induced cytochrome c release, although sorafenib alone at doses higher than 10 μM triggered cytochrome c translocation in O/N experiments (not shown here).

Since mitochondrial cytochrome c release frequently triggers caspase-dependent cell death through activation of executor caspases, we measured caspase-3 levels compared to TNF plus cycloheximide. Sorafenib increased caspase-3 activity modestly in hepatoma cells, that was not further enhanced by PDMP addition (Figure 6D), despite the induction of cytochrome c release and cell death. Notably, sorafenib/PDMP-induced cell death was not blocked by pre-incubation with a pan caspase inhibitor ZVAD (Figure 5D), at doses previously verified to block Fas-induced apoptosis [17], further suggesting that the mechanism involved in PDMP sensitization is caspase-independent.

Elevation of glycolysis and enhanced autophagy may cooperate to protect cells from caspase independent cell death [28], preserving viability even after decline of MMP. Release of mitochondrial intermembrane proteins such as cytochrome c induces a rapid loss of activity in respiratory complexes jeopardizing mitochondrial functionality, and leading to bioenergetic crisis and cell death [34]. To test if GCS inhibition triggers this mechanism, we determined changes in ATP concentration on sorafenib/PDMP exposed cells. While neither sorafenib nor GCS inhibition reduced ATP levels in Hep3B cells, the combination sorafenib/PDMP depleted ATP levels (Figure 6E), suggesting the induction of mitochondrial collapse by GCS inhibition in sorafenib-treated cells. To discard that this effect was caused by a decline in the number of mitochondria as a consequence of the treatments, we examined for changes in the mitochondrial DNA copy number in cells [35]. Despite the mitochondrial dysfunction observed after 6 hours following sorafenib/PDMP administration, no alteration in mitochondrial DNA amount was detected indicating that a decrease in mitochondrial mass was not the reason for the mitochondrial alteration (Suppl. Fig. 7).

Moreover, although most tumors exhibit a preferential switch to glycolysis, ceramide may reduce GAPDH expression targeting the “Warburg effect”, as observed in melanoma cells [36]. However, sorafenib did not change GAPDH expression regardless of the presence of PDMP (Suppl. Fig. 8), suggesting a mitochondrial contribution in the ATP decline caused by the PDMP/sorafenib cotreatment. Moreover, GCS inhibition in sorafenib-treated cells caused significant nuclear condensation (12.7±2.5, 8 hours), detected by Hoechst staining (Figure 6F), compared to sorafenib (2.3±0.7%) or PDMP (1.6±0.7%) alone, without evidences of fragmented nuclei on later times of incubation (data not shown). Apparently, since executioner caspases require for full apoptotic death a non-oxidative environment and an operational ATP production, in sorafenib/PDMP treated cells, despite cytochrome c release, caspase activation is blocked forcing the cell to die by a caspase-independent mechanism after mitochondrial collapse. However, to better characterize this event a complete bioenergetic study with a flux analyzer would be required.

### Hepatoma cell lines exhibit acquired sorafenib-resistance and high GCS expression after long-time exposure to sorafenib

**Figure 7:**
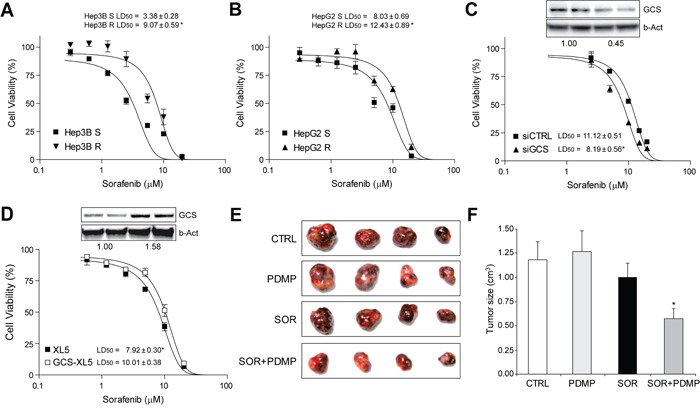
Sorafenib-resistant hepatoma cells were re-sensitized to sorafenib exposure by GCS targeting *in vitro* and in tumor mouse model **A.** and **B.** dose-response analysis by MTT in Hep3B and HepG2 cells after long-term drug exposure. **C.** Cell viability of Hep3B R cells after transfection with siRNA against GCS (siGCS) or control (siCTRL) and sorafenib exposure. Representative image of protein levels in upper panel. *, p<0.05 vs. siCTRL Hep3B cells. **D.** Hep3B cells transfected with vector control (PCMV6-XL5) or GCS-PCMV6-XL5, with protein levels shown in upper panel, were exposed to sorafenib and cell viability determined. *, p<0.05 vs. sorafenib-sensitive (S) hepatoma cells. **E.** and **F.** Representative image of sorafenib-resistant HepG2 subcutaneous tumors after 4 weeks of treatment with vehicle (CTRL), sorafenib and/or GCS inhibitor (PDMP), and graphical representation of tumor volumes (CTRL, n=6; PDMP, n=5; SOR, n=12; SOR+PDMP, n=11). *, p<0.05 vs. sorafenib-treated (SOR) tumors.

The systemic treatment with sorafenib in patients with advanced HCC results in limited survival benefits suggesting the existence of primary and acquired drug resistance mechanisms [4–6]. To evaluate if GCS overexpression may participate in sorafenib-resistant phenotype, Hep3B and HepG2 cells were grown during 12 months in the presence of sorafenib (0-5 μM) leading to sorafenib resistance (Figure 7A-7B). Of note, before MTT assays, hepatoma cells chronically exposed to sorafenib were maintained in culture medium without sorafenib for a week before assays. Moreover, hepatoma resistant cells displayed almost no reduction in ERK signaling after sorafenib exposure, in opposition to sensitive cells, while exhibiting resistance for more than one month after sorafenib withdrawal and cross-resistance to other chemotherapeutic agents, such as doxorubicin (data not shown). Afterwards, we checked for alterations in the sphingolipid metabolism after long-term sorafenib administration in hepatoma cells. While short-time sorafenib addition stimulated mRNA changes in several sphingolipidic enzymes (Table 1), Hep3B cells with acquired sorafenib resistance exhibited modifications only in very specific sphingolipidic proteins (Table 2), most prominently in GCS expression. An effect also observed in HepG2 resistant cells (Suppl. Table 2). To validate if GCS expression could play a role in sorafenib resistance, we reduced GCS levels by RNA interference. Sorafenib-resistant Hep3B cells (Hep3B R cells) transfected with siRNA against GCS displayed increased sensitivity upon sorafenib administration (Figure 7C). Moreover, pharmacological inhibition of GCS with PDMP was effective in reducing sorafenib resistant in Hep3B R cells dose-dependently (Suppl. Fig. 9). Similar results were also observed in HepG2 resistant cells (Suppl. Fig. 10). To further verify this observation, we overexpressed GCS in Hep3B cells before exposure to sorafenib (Figure 7D). GCS-transfected Hep3B cells displayed reduced sorafenib-induced cell death, suggesting that GCS mediates, at least partially, sorafenib-acquired resistance in hepatoma cells.

**Table 2:** mRNA levels of main sphingolipidic enzymes in sorafenib-resistant and sensitive Hep3B cells

	Hep3B S	Hep3B R
ASMase	1.00±0.25	0.97±0.10
NSMase	1.00±0.22	1.12±0.22
ACDase	1.00±0.11	1.04±0.37
NCDase	1.00±0.18	0.98±0.23
CerS2	1.00±0.15	1.20±0.19
CerS4	1.00±0.27	1.05±0.29
GCS	1.00±0.09	4.28±0.38 *
SPT	1.00±0.16	1.17±0.13

Ceramide-related enzymes were analyzed by RT-PCR in Hep3B cells that exhibit sorafenib resistance after long-term exposure to sorafenib (Hep3B R) or vehicle (Hep3B S). (n=3).

* p<0.05 vs. control.

Therefore, since our results indicated that GCS overexpression could contribute to the inefficacy of sorafenib-therapy, we addressed if enhanced expression of GCS also occurs in tumors from HCC patients. To do so, we measured GCS mRNA levels in a human cDNA array from normal liver tissue or individuals with different HCC staging. Our results did not detect changes in GCS levels depending on the HCC stage (Suppl. Fig. 11), probably indicating a relevant role of GCS in cancer-resistance rather than in tumor progression or invasiveness.

### Sorafenib-resistant HepG2 xenografts tumors displayed sorafenib sensitivity after GCS inhibition

Enhanced metastatic potential of hepatoma cells with sorafenib resistance has been previously reported in an orthotopic HCC model [37]. In our experiments, we did not detected differences in terms of multiplicity or engraftment between sorafenib-resistant HepG2 tumors compared to mice inoculated with parental HepG2 cells. Once developed, animals were randomly divided in groups and treated with sorafenib or vehicle in combination with GCS inhibitor PDMP. Our results verified tumor sorafenib resistance during the study, while the combined therapy (PDMP plus sorafenib) significantly reduced the growth of resistant tumors (Figure 7E-7F). Of note, GCS inhibition alone did not affect tumor volume (Figure 7F), and did not cause any detectable liver damage alone or combined with sorafenib (data not shown). In conclusion, GCS antagonism restored tumor sensitivity to sorafenib *in vivo*, suggesting GCS targeting as an interesting strategy against sorafenib-acquired resistance.

## DISCUSSION

Sorafenib is the only approved systemic drug recommended for HCC patients with recurrence after resection/ablation or diagnosed at advanced stages [38, 39]. Although other molecular therapies, tested head-to-head versus sorafenib, are explored in phase III clinical trials, none of them have achieved superior results [4–6]. Therefore, despite its limitations, sorafenib is so far the best option for advanced HCC and the first drug able to disclose a weakness in HCC biology likely due to its targeting of multiple pathways. Further research is needed to identify novel molecular targets, but efforts to explore the efficacy of combination therapies with sorafenib should not be obviated. In this sense, our data reveals that ceramide metabolism is activated during sorafenib administration, making plausible strategies in HCC treatment aimed to increase cytotoxicity via ceramide accumulation, by targeting specific ceramide-degrading enzymes such as GCS. Importantly, GCS is overexpressed in sorafenib resistant hepatoma cells after long term exposure, pointing to GCS induction as a specific mediator of sorafenib resistance that provides a potential target for cancer therapy.

Most common mutations in HCC, such as p53 and beta-catenin, are undruggable, therefore, suggesting the need to exploit the use of proteins with well-known inhibitors such as GCS to improve HCC management. In this sense, PDMP [40], is a well characterized GCS inhibitor and has served as a basis of novel GCS inhibitors, which are now tested in clinical trials. Particularly, eliglustat tartrate, a PDMP derivative designed for the treatment of lysosomal diseases [41], have recently reached FDA approval for Gaucher's disease patients [42]. Obviously, confirmation in preclinical models would be required, but it is tempting to speculate about positive results with immediate medical application.

Compounds that potentiates sorafenib efficacy are important since validate potential targets, and also provide clues for other products acting in the same metabolic pathway or in the same subcellular location. In this sense, our work points to the mitochondria as the organelle where sorafenib toxicity is triggered by PDMP addition. Several reports indicate that sorafenib interferes with the mitochondrial respiratory machinery, inducing loss of membrane potential and ROS production. However, our data shows that sorafenib-treated cells maintained mitochondrial integrity, without any loss of cytochrome c, and with relatively normal ATP levels for several hours. In fact, autophagy induction and the capacity of tumor cells of generating ATP from extra-mitochondrial source via glycolysis (Warburg effect) are adaptive mechanisms that allow tumor recovering after strong mitochondrial damage, sometimes from only a small fraction of surviving mitochondria [43]. It is conceivable that sorafenib is acting similarly in the absence of other mitochondrial-damaging stimuli, being insufficient to cause death in most hepatoma cells. In fact, recent data indicates that the mitochondrial damage induce by sorafenib is accompanied by progressive glycolytic reprogramming to help cells to survive under energetic stress [44]. Regarding this point, we cannot rule out that GCS inhibition may be also blocking the glycolytic pathway of ATP generation, such as observed in chronic lymphocytic leukemia treated with nanoliposomal ceramide [36]. However, we detected no reduction in GADPH activity after PDMP/sorafenib treatment. In line with these finding, our data support a pathway in which GCS inhibition leading to increase ceramide levels targets mitochondria, inducing cytochrome c release, loss of ATP and energetic collapse, making hepatoma cells incapable of recovery and destined to die.

Numerous publications have shown how ceramide accumulation perturbs the mitochondrial integrity [13, 16, 14, 31–33], similarly to our observations in sorafenib-treated hepatoma cells. In particular, changes in sphingolipid composition of the mitochondrial membranes seem to alter the specific lipid milieu required for Bak/Bax activation modifying the cell death induced by BH3-only proteins [32, 33]. Therefore, it is plausible that molecules, such as specific Bcl-2 inhibitors, could elude the incomplete MMP induced by sorafenib by breaking the resistance at the point of Bax or Bak activation, as observed in the PDMP/sorafenib combination. In line with this, Bcl-xL inactivation (ABT-737) in combination with sorafenib, that down-regulates Mcl-1 expression specifically in tumor cells, efficiently induced cell death in hepatoma cells [45]. In fact, a recent report has shown in vinorelbine-resistant lung adenocarcinoma cells an increase in GCS activity which was associated with induction of Bcl-xL-mediated cell survival [46]. Interestingly, this is not the only link established between ceramide metabolism and Bcl-2 protection. A recent publication proposes a feed-forward model by which BAK activation by chemotherapeutic drugs, and particularly by BH3 mimetics, leads to elevated ceramide levels resulting in synergistic channel formation by ceramide metabolites and BAX/BAK. Certainly, if this mechanism is induced by the combination of sorafenib and Bcl-2 mimetics is a point that deserves further investigation [47]. In fact, it is possible that other mitochondrial interfering molecules may potentiate sorafenib efficacy maybe due to the dependence on mitochondrial biogenesis of cancer stem cells survival [48]. Accordingly, our results point to GCS targeting as an interesting approach to increase sorafenib efficacy in HCC management, and support strategies aiming mitochondria to improve sorafenib therapy.

## MATERIALS AND METHODS

### Cell culture and conditioned medium preparation

Human liver tumor cell lines Hep3B, PLC and HepG2 (European Collection of Animal Cell Cultures (ECACC)) were grown in DMEM (10% FBS) at 37°C and 5% CO_2_. To generate sorafenib-resistant hepatoma cells, freshly thawed Hep3B and HepG2 cells were cultured with 1 μM of sorafenib and, after a month, the concentration slowly increased by 0.5 μM per month (up to 5 μM). After 8 to 10 months, two sorafenib-resistant cell lines, termed HepG2 R and Hep3B R, were obtained. The LD50 of the cells to sorafenib was determined in 96-well plates, routinely for 24 hours and cell viability measured by MTT assay.

### qPCR and Immunoblot analysis

Total DNA isolated with and total RNA with TRIzol reagent were analyzed with SensiFAST SYBR One-Step Kit (Bioline. Ecogen, Barcelona, Spain) following the manufacturer's instructions, as detailed in Supplemental Methods. Western blots were performed as indicated in Supplemental Methods.

### RNA interference and GCS overexpression

HepG2 and Hep3B cells were transfected with siRNAs, designed to knockdown gene expression of GCS, ACDase or control (siGCS, sc-45404; siACD, sc-105032; siCTRL, sc-37007, Santa Cruz Biotechnologies), or with GCS-expressing or PCMV6-XL5 control vectors (Origene, Rockville, MD, USA). RNA silencing or GCS overexpression were verified by western blot and qPCR, as detailed in Supplemental Methods.

### Biochemical analysis

Cell viability, clonogenic assays, Hoechst staining, caspase-3 activity, mitochondrial membrane potential (MMP), reactive oxygen species (ROS) production [17], mitochondrial DNA content, mitochondrial Complex I activity, GAPDH expression and ATP levels were analyzed as explained in Supplemental Methods. Ceramide determination after [^14^C]palmitic acid labeling, and GCS/ACDase activities were performed as previously described [13, 18], and detailed in Supplemental Methods.

### Tumor animal model

All animal procedures were performed according to protocols approved by the Animal Experimentation Ethics Committee from the University of Barcelona. For subcutaneous tumor model, male Swiss nude mice, 5-6 week old, were kept under pathogen-free conditions with free access to standard food and water. HepG2 cells (5×10^6^) or Hep3B cells (2.5×10^6^) were injected subcutaneously into the flanks of mice in 200 μL DMEM without FBS, as previously reported [13, 19]. Treatment with GCS inhibitor 1-phenyl-2-decanoylamino-3-morpholino-1-propranol (PDMP) or vehicle (saline solution) was delivered i.p. daily, while sorafenib was administered via oral gavage at a dose of 80 μg/g body weight for 21 days. Tumors were measured periodically with a vernier caliper, and the volume was calculated as length×width^2^×0.5.

### Immunohistochemical staining

Tumors were fixed and 5-μm sections were prepared following standard procedures. The antibodies used were mAb anti-PCNA antibody (PC10) (1:200, sc-56, Santa Cruz) and anti-CD34 (1:100, sc-18917, Santa Cruz). The slices were examined with a Zeiss Axioplan microscope equipped with a Nikon DXM1200F digital camera. PCNA index was quantified in four randomly selected fields from each animal, and CD34 positive areas analyzed using ImageJ software. Apoptotic cells with fragmented nuclei were detected in paraffin samples using TUNEL labeling containing fluorescein-dUTP and -dNTPs (TUNEL Label Mix, Roche). TUNEL positive cells were observed and quantified using a NIKON Eclipse E-100 microscope.

### Statistical analyses

Results are expressed as mean ± standard deviation and n=3, unless indicated. Statistical comparisons were performed using unpaired 2-tailed Student's t test or 1-way ANOVA followed by Newman-Keuls Multiple Comparison Test (GraphPad Prism). A *P* value less than 0.05 was considered significant.

## SUPPLEMENTARY DATA, FIGURES AND TABLES



## Abbreviations

ACDase: acid ceramidase
ASMase: acid sphingomyelinase
GCS: glucosylceramide synthase
CerS: ceramide synthase
HCC: hepatocellular carcinoma
MDR: multidrug-resistance
MMP: mitochondrial membrane potential
PDMP: 1-phenyl-2-decanoylamino-3-morpholino-1-propranol
NOE: N-oleoylethanolamine
PCNA: proliferating cell nuclear antigen
ROS: reactive oxygen species
SK1: sphingosine kinase 1
SPT: serine palmitoyl transferase
WT: wild type
