# Supplementary material for: Targeting glucosylceramide synthase upregulation reverts sorafenib resistance in experimental hepatocellular carcinoma
Source: Oncotarget. 2016 Jan 22;7(7):8253–67. doi: 10.18632/oncotarget.6982 (PMC4884990; doi:10.18632/oncotarget.6982)
Supplement: Supplementary file 1 [file oncotarget-07-8253-s001.pdf]

SUPPLEMENTARY DATA

TABLE OF CONTENTS

Supplemental Methods page 1
Reagents page 1
SDS-PAGE and immunoblot analysis page 1
RNA isolation and real time RT-PCR page 1
MTT assay page 2
Crystal Violet, Caspase-3 and JC1 assays page 2
Hoechst staining, Reactive Oxygen Species and ATP assays page 2
GAPDH, Mitochondrial DNA content and Complex I activity page 3
Ceramide quantification and GCS assay page 3
ACDAse assay and cDNA Array page 4
Supplemental Figures page 5

SUPPLEMENTAL METHODS

Reagents

Dulbecco's Modified Eagle's Medium (DMEM), trypsin-EDTA, penicillin-streptomycin and dimethyl sulfoxide (DMSO), MTT (3-(4,5-dimethylthiazol-2-yl)-2,5-diphenyl tetrazolium bromide) (M2128), Hoechst 33258 (B1155), Crystal Violet (C0755), N-Oleoylethanolamine (NOE) (O0383), Imipramine (I0899) and DCF (D6883) were purchased from Sigma-Aldrich (St. Louis, MO). All tissue culture-ware was from Nunc (Roskilde, Denmark). Biotin Blocking System, peroxidase substrate (DAB) and peroxidase buffer were from DAKO (Glostrup, Denmark). Aquatex was from Merck (Darmstadt, Germany). The ABC kit was from Vecstain (Burlingame, CA). Proteinase inhibitors were from Roche (Madrid, Spain). ECL western blotting substrate was from Pierce (Thermo Fisher Scientific, Rockford, IL). UGCG siRNA (h) (sc-45404), Acid Ceramidase siRNA (h) sc-105032 and scrambled controls were purchased from Santa Cruz Biotechnology (Dalla, TX); as well as DL-threo-1-Phenyl-2-decanoyl amino-3-morpholino-1-propanol (Dl–threo-PDMP, Hydrochloride) (sc-203030), C-6 NBD Ceramide (N-Hexanoylsphingosine) (sc-204661), C-12 NBD Ceramide (N-Palmitoylsphingosine) (sc-205232), HA14-1 (sc-205911), Z-VAD-FMK (sc-3067), 3 Methyladenine (3-MA) (sc-205596) and Bafilomycin A1 (sc-201550). One Shot® TOP10 chemically competent cells, Lipofectamine2000 (11668-027), Lipofectamine3000 (L3000008), Novex Sharp Pre-stained Protein Standard (LC5800) and JC-1 (T-3168) were from Invitrogen Life Technologies (Carlsbad, CA) Sorafenib (BAY 43-9006, Nexavar) is manufactured by Bayer. C6- Ceramide (N-Hexanoylsphingosine) (BML-SL-110-0005); C16-Ceramide (N-Palmitoylsphingosine) (BML-SL-115-0005); Myriocin (BML-SL226-0005) were from Enzo Life Sciences (Farmingdale, NY).

SDS-PAGE and immunoblot analysis

Cell lysates were prepared in RIPA buffer plus proteinase inhibitors. Samples containing 10 to 30 µg were separated by 8-10% SDS-PAGE. Proteins were transferred to nitrocellulose membranes, blocked in 8% nonfat milk for 1h at room temperature, and incubated overnight at 4°C with the primary antibodies:

BECLIN-1 (SantaCruz, sc-11427) 1:250 rabbit
Mcl-1 (S-19) (SantaCruz, sc-819) 1:250 rabbit
Bcl-2 (N-19) (SantaCruz, sc 492) 1:250 rabbit
P62/SQSTM1 (D-3) (SantaCruz, sc-28359) 1:250 mouse
pAKT 1/2/3 (ser 473)-R (SantaCruz, sc-7985-R) 1:250 rabbit
AKT 1/2 (N-19) (SantaCruz, sc-1619) 1:250 rabbit
MAP LC3β (H-50) (SantaCruz, sc-28266) 1:250 rabbit
MAP Kinase diphosphorylated (pERK1/2) (Sigma, M 8159) 1:1000 mouse
MAP Kinase (ERK1/2) (Sigma, M 5670) 1:10000 rabbit
GCS/UGCG (Proteintech, 12869-1-AP) 1:1000 rabbit
ACDase (BD Biosciences, 612302) 1:1000 mouse
Cytochrome C (SantaCruz, sc-1356) 1:250 mouse

RNA isolation and real time RT-PCR

Total RNA was isolated with TRIzol reagent. Real-time RT-PCR was performed with SensiFAST SYBR One-Step Kit (Bioline. Ecogen, Barcelona, Spain) following the manufacturer's instructions. The primers sequences used were:

Acid Ceramidase: human ACDase, Fw 5' CTACCCAAGTCTCAGCGCGCTT 3', Rv 5' GCACCTCTGTACGTTGGTCCTGAA 3' (GenBank # NM_004315);

Glucosylceramide synthase: human GCS, Fw 5' GCCTTGGAGGGAATGGCCGTC 3' Rv 5' GAGAGACACCTGGGAGCTTGCTA 3' (GenBank # NM_003358);

Acid Sphingomyelinase: human ASMase Fw 5' GTCTCCGCCTCATCTCTCTC 3' Rv 5' GCACTTTGTCTCCTCGATCC 3' (GenBank # NM_000543)

Ceramide Synthase 2 : human CerS2 Fw 5' ACCCGAGCAGACGGAGTACACGG 3', Rv 5' ACCCGAGCAGACGGAGTACACGG 3' (GenBank # NM_181746)

Serine Palmitoil Synthase: human SPT Fw 5’ CGTGGGGACTTGTGGACCCA 3’, Rv 5’ AGCACTGGCTATGGTGGCAAATCC 3’ (GenBank # NM_006415)

Sphingosine Kinase: human SphK Fw 5’ GGGCGCTCCAGTCCCTCAGA 3’, Rv 5’ GTCGGTCCGGTTTGCTGGGG 3’ (GenBank # NM_021972)

β-Actin: human β-Actin Fw 5’ GGACTTCGAGCAAGAGATGG 3’, Rv 5’ AGGAAGGAAGGCTGGAAGAG 3’ (GenBank # NM_001101)

18S Ribosomal RNA: human 18S Fw 5’ CCGAAGATATGCTCATGTGG 3’, Rv 5’ TCTTGTACTGGCGTGGATTC 3’ (GenBank # NM_022551.2)

MTT assay

Cytotoxicity of sorafenib and other reagents were determined by the MTT (3-(4,5-dimethylthiazol-2-yl)-2,5-diphenyl tetrazolium bromide) assay. About 3×10^4 cells per well were plated into a 96-well plate and incubated in 5% CO2 at 37°C. Stock solution (5 mg/ml) of sterile filtered MTT in phosphate buffered saline (PBS) pH 7.4 was added 1/10 to each well. After 2 h of incubation at 37°C, unreacted dye was removed by aspiration, the insoluble formazan crystals were dissolved in 100 µl/well of solubilization/stop solution (1-propanol) and measured spectrophotometrically in an ELISA reader (Titertek Plus MS 212, ICN, Eschwege, Germany) at a wavelength of 570 nm, and 630 nm (reference). The spectrophotometer was calibrated to zero absorbance using 1-propanol. The net A570 - A630 was taken as the index of cell viability. The net absorbance change taken from the wells of untreated cultured cells was used as the 100% viability value. The relative cell viability (%) related to control wells was calculated by the formula 100X [(A570-A630) sample/(A570-A630)control].

Crystal violet assay

About $5x10^4$ cells per well were plated into a 12-well plate and incubated in 5% CO2 at 37°C. After four days of treatments, cells were fixed for 5 min. with 10% formalin, stained for 30 minutes and washed twice with tap water. After draining, plates were inverted for 5 minutes and photographed.

Caspase-3 activity determination

Cells were treated with different extracellular factors, media was removed and cells were scrapped in a buffer containing 120 mM NaCl, 50 mM Tris-HCl, pH7.4, 0.5 % Igepal, 2 mM ethylene glycol-bis-(2-aminoethyl ether) tetraacetic acid, and 50 µM PMSF, incubated at 4°C for 15 minutes with shaking, and spun down at 12,000 g at 4°C for 15 minutes. Caspase activity was assayed with 200 µg of cell lysate and 1.25 ml of assay buffer containing 100 mM NaCl, 10% sucrose, 0.1% (3-[(3-cholamidopropyl)dimethylammonio]-1-propanesulfonate (CHAPS), and 10 mM DTT, pH 7.4, by the release of 7-aminomethyl coumarin (AMC) from 40 nmol of Ac-DEVD-AMC (Calbiochem). Fluorescence was continuously recorded with emission at 460 nm and excitation at 380 nm. A unit of caspase-3 activity is the amount of active enzyme necessary to produce an increase in 1 fluorescence unit in spectrofluorimeter. Results are usually represented as Arbitrary Unit/h/µg protein. Data are expressed as fold induction versus control.

Analysis of mitochondrial transmembrane potencial by JC-1 staining assay

Cells were loaded with C5,5′,6,6′-tetrachloro-1,1′,3,3′-tetraethylbenzimidazolyl-carbocyanine iodide (JC-1), a cationic dye that exhibits potential-dependent accumulation in mitochondria, indicated by a fluorescence emission shift from green (~525 nm) to red (~590 nm). Consequently, mitochondrial depolarization is indicated by a decrease in the red/green fluorescence intensity ratio. To evaluate the mitochondrial depolarization induced by drug treatment, we plated 5×10^5 Hep3B cells in 6 well plates. The following day cells were treated as indicated in each experiment. After the treatment, cells were stained for 20 minutes in medium containing JC-1 at the final concentration of 5 µg/ml. After removal of JC-1, cells were washed once by PBS. Fluorescence is measured in a Microplate Fluorescence Reader using an exciting wavelength of 550 (535) nm for red and 485 nm for green, and an emission wavelength of 600 (590) nm and 535 (530) nm, respectively. Results are calculated as a ratio of red fluorescence divided by green fluorescence. The ratio of red to green fluorescence is decreased in dead cells and in cells undergoing apoptosis compared to healthy cells.

Hoechst staining

About 5×10^4 cells per well were plated into a 12-well plate and incubated in 5% CO_2 at 37°C. After six hours of treatments, 1/1000 of a Hoechst 33258 (10 mg/ml stock solution) was added and incubated for 30 min. Cells were washed twice with regular medium. Images were taken under UV light and 12 random fields for each condition were counted to establish the percentage of cells with condensed nucleus.

Reactive Oxygen Species (ROS) measurement

In order to measure the intracellular content of ROS, we used fluorimetric method. Cells were plated in 12-well plates, and after the treatment they were washed with PBS and incubated with 10 µM 2′, 7’-dichlorodihydrofluorescein diacetate (H2DCFDA) in HBSS during 30 minutes at 37°C. The color was incorporated into the cells and converted into 2′,7′-dichlorofluorescein (DCF) when oxidized by hydrogen peroxide. Fluorescence is measured in a Microplate Fluorescence Reader using an exciting wavelength of 485 nm and an emission wavelength of 520 nm. Results are calculated as fluorescence units per µg protein and expressed as percentage of control.

ATP determination

To determine the intracellular ATP content, we used a bioluminescent assay from Sigma-Aldrich. In short, hepatoma cells were plated in 96-well plates. After the treatments, culture medium was removed, 90 µL of ATP reagent added to each well and the plate tapped briefly to mix. After 1 minute incubation at room temperature, light emission was determined using a luminometer and images of the plate taken with ImageQuant LAS 4000 equipment (GE Healthcare Bio-Sciences AB, Uppsala, Sweden). Results are calculated as light units per µg protein and expressed as percentage of control.

GAPDH activity assay

To determine the intracellular GAPDH activity we used a spectrophotometric kit from Biovision (#K680-100). In short, through this assay GAPDH catalyzes conversion of GAP into 1, 3-Bisphosphate Glycerate and an intermediate, which reacts with the developer to form a colored product (maximal Abs. 450 nm). Results, after standard comparison, are expressed as GAPDH µUnits per ml and minute.

Mitochondrial DNA content

Total DNA was extracted using QuickExtract™ DNA Extraction Solution (Epicentre, Madison, WI) following manufacturer’s instructions and changes in mitochondrial DNA copies calculated by comparison with nuclear DNA content in the same samples, as suggested by Phillips *et al*. Specific primers for qPCR used were:

Mitochondrial DNA: mtMinArc Fw 5’ CTAAATAGCCCACACGTTCCC 3’, Rv 5’ AGAGCTCCCGTGAGTGGTTA 3’ (GenBank # NC_012920)

Nuclear DNA: β2M Fw 5’ GCTGGGTAGCTCTAAACAATGTATTCA 3’, Rv 5’ CCATGTACTAACAAATGTCTAAAATGGT 3’ (GenBank # NT_010194.17)

Complex I activity

For the analysis of mitochondrial OXPHOS Complex I enzyme activity from hepatoma protein extracts we used the Complex I Enzyme Activity Dipstick Assay Kit (ab109720) following the manufacturer’s instructions. In brief, 20 µg of protein extract from treated cells were loaded onto a dipstick for each sample. Individual dipsticks were incubated with activity buffer solution containing NADH as a substrate and nitrotetrazolium blue (NBT) as the electron acceptor, and developed for 45 minutes. After that, bands were photographed and quantified.

[^{14}C] labeled ceramide quantification by TLC

Hepatocellular carcinoma cells Hep3B were cultured in Dulbecco’s modified Eagle medium supplemented with 10% heat inactivated fetal bovine serum. Cells were cultured and labeled with [^{14}C] palmitic acid (2 µCi/ml) (Amersham, Piscataway, NJ) for 24h. After the treatment with Sorafenib/PDMP/NOE, cells were washed three times with phosphate buffered saline, scraped off the dish and centrifuged at 6000 rpm for 5 min. Total lipids were extracted from the pellets with 1 ml chloroform/methanol (2:1 by volume), 4h. Ceramide was resolved in LK6D gel 60A TLC plates using chloroform/acetic acid (90:10). Ceramides were separated from the other lipids by thin layer chromatography using diethylether/methanol (99:2, v:v) as the developing system. Two separate spots of radiolabeled lipids were detected on the chromatogram by radioactive scanning.

GCS enzyme assay

The hepatocarcinoma cells were seeded in 6 well plates at $5{\times}10^5$/well. After treatment the samples were scraped off the dish with TE plus 0.1% TritonX-100 pH=7.4, vortexed, centrifuged at 12000 rpm for 5 minutes. Supernatant protein (150 mg/ml) was added to a mix of C6-NBD (1µL/sample), UDP-Glucose (5µL/sample) and assay buffer Phosphate 0.1% Triton pH=7.8, for the final volume of 250uL per each sample. Samples were incubated for 1h at 37°C, added 750uL of chloroform:methanol (2:1, v:v), vortexed and centrifuged at 5000 rpm for 5 min. The top (aqueous) layer of each tube is then removed by aspiration and discarded. The bottom layer was transferred to new tubes and dried with heat at the speed-vac. Samples were then redissolved in 50uL of spotting solvent, methanol:chloroform (1:1, v:v), and run on Silica 60 thin-layer chromatography plates in chloroform:methanol:ammonia (90:20:0.5 mL) along

with appropriate C6-NBD-lipid standards. The fluorescent lipids on the plates were visualized by UV illumination and quantified using an Alphaimager 2200 Analysis System (Alexandria, VA) and results confirmed in a Perkin Elmer LS50B luminescence spectrometer set at 475 nm for excitation and 525 nm for emission.

ACDase enzyme assay

The cells were seeded in 6 well plates at $5x10^5$ cell/well concentration. After treatments, the samples were lysed in an acidic buffer (pH 4.5) consisting of 50 mM sodium acetate, 5 mM magnesium chloride, 1mM EDTA, and 0.5% TritonX-100, and samples centrifuged at 12000rpm for 5 minutes. Supernatant protein (150 µg) was incubated for 1h at 37°C in the assay buffer (pH 4.5) with 0.2% Igepal-CA 630, 250 mM sodium acetate, and 150 µM of C12-NBD (1µL/sample), for the final volume of 250 µL per each sample. Then, 750 µL of chloroform:methanol (2:1, v:v) was added to each tube, vortexed, and centrifuged at 5000 rpm for 5 min. The top (aqueous) layer of each tube was removed by aspiration and discarded. The bottom layer was transferred to new tubes and dried with heat at the speed-vac. Samples were then redissolved in 50uL of spotting solvent, chloroform:methanol (1:1, v:v), and run on Silica 60 thin-layer chromatography plates in chloroform:methanol:ammonia (90:20:0.5) along with appropriate C12-NBD-lipid standards. The fluorescent lipids on the plates were visualized by UV illumination and quantified using an Alphaimager 2200 Analysis System (Alexandria, VA) and results confirmed in a Perkin Elmer LS50B luminescence spectrometer set at 475 nm for excitation and 525 nm for emission.

cDNA array

TissueScan™ cDNA Array (Liver Cancer cDNA Array I, Origene) was used to quantify GCS levels in tumor and normal tissues. Tissue cDNAs of each array are synthesized from high quality total RNAs of pathologist-verified tissues, normalized and validated with ß-actin in two sequential qPCR analyses, and provided with clinical information and QC data. Our array contained cDNA from 48 samples covering 8-normal, 7-Stage I, 8-II, 8-IIIA, 3-IV and 13- Liver Lesions in identical plates (LVRT101).

SUPPLEMENTARY FIGURES AND TABLES

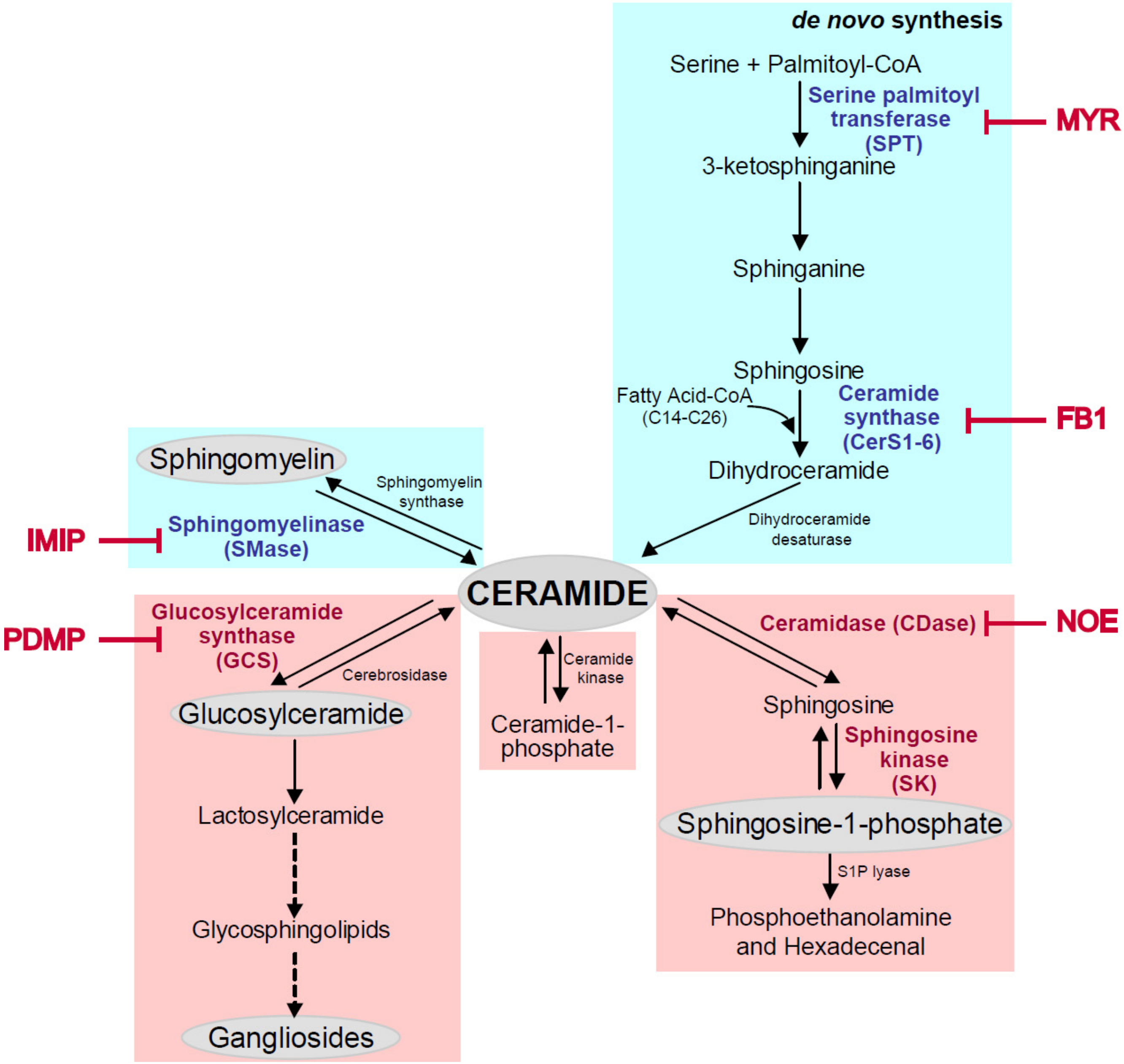

Supplementary Figure S1: Schematic representation of main cellular pathways involved in ceramide metabolism in the liver. Ceramide is mainly generated (in blue): i) *de novo* after condensation of serine with palmitoyl-CoA by the action of the serine palmitoyl transferase (SPT). Afterwards, the long-chain base sphingosine is attached to fatty acyl CoAs of different length through ceramide synthase (CerS1-6) activity; ii) from breakdown of sphingomyelin upon activation of sphingomyelinases (SMases). Ceramide modification to less toxic species occurs (in red) by: a) glycosylation (GCS) to form glycosphingolipids, such as gangliosides; b) transformation to sphingosine-1-phosphate via ceramidase (CDase) and sphingosine kinase (SK) activation; c) phosphorylation by ceramide kinase.

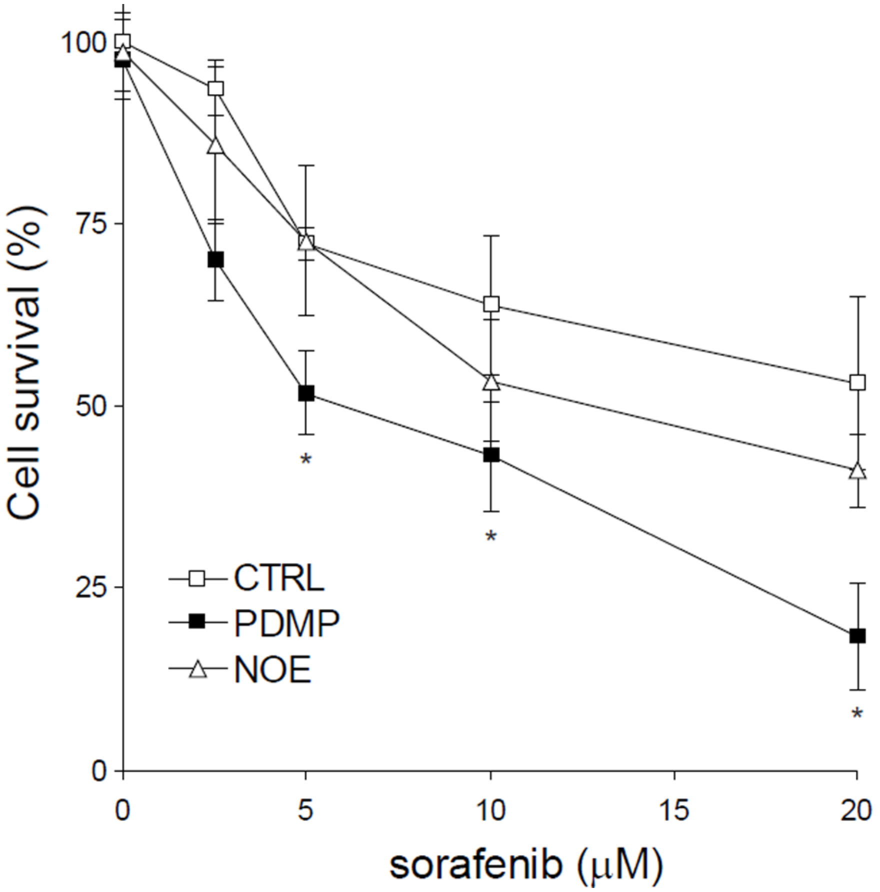

Supplementary Figure S2: Cell viability after sorafenib administration in HepG2 cells treated with vehicle (CTRL), GCS inhibitor (PDMP) or ACDase inhibitor (NOE) during 16 hours. (n=2). *, $p<0.05$ vs. CTRL HepG2 cells.

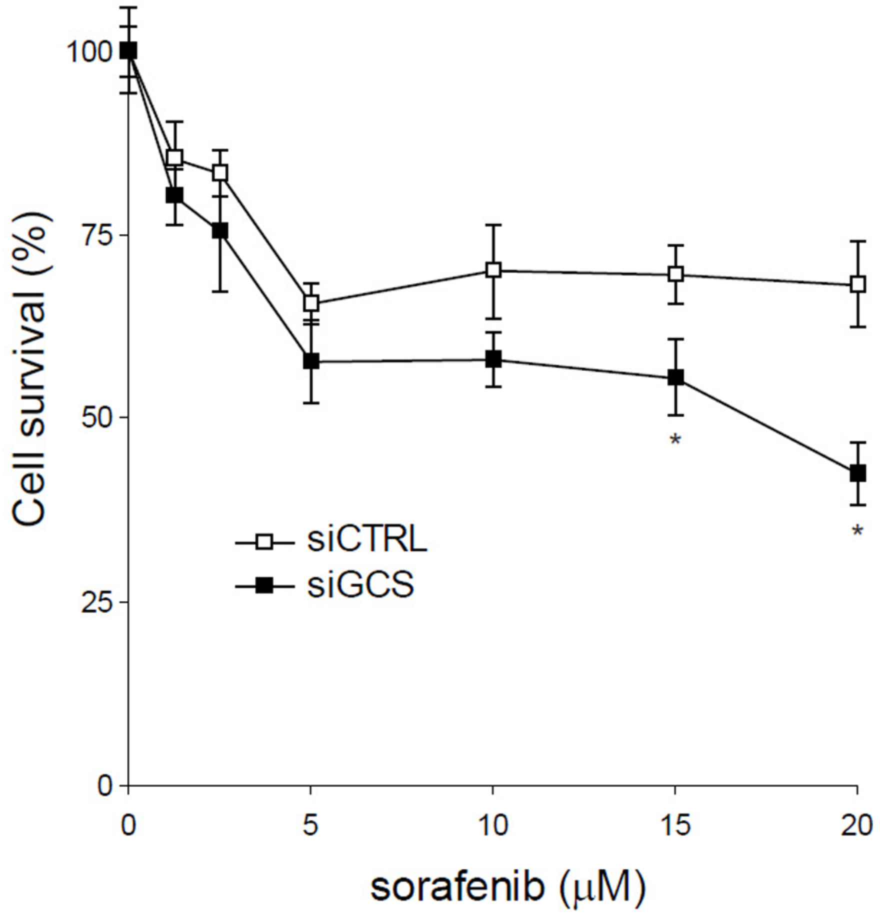

Supplementary Figure S3: Cell viability after sorafenib administration (16 h) in HepG2 cells transfected with siRNA control or against GCS 48 h before. (n=2). *, $p<0.05$ vs. siCTRL HepG2 cells.

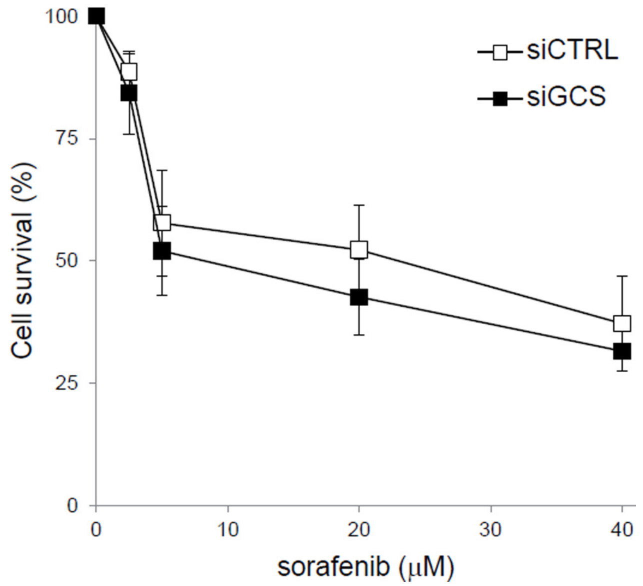

Supplementary Figure S4: Cell viability after sorafenib administration (16 h) in PLC cells transfected with siRNA control or against GCS 48 h before. (n=3). *, $p<0.05$ vs. CTRL PLC cells.

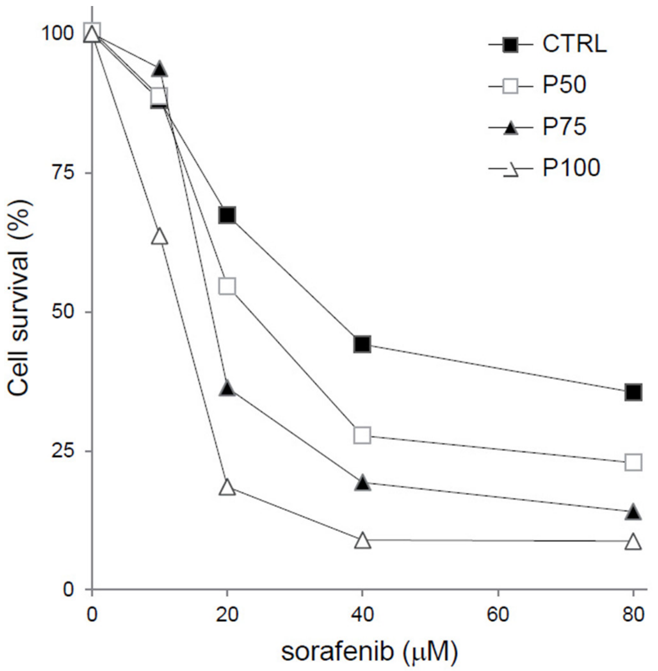

Supplementary Figure S5: Representative experiment measuring cell viability after sorafenib administration in PLC cells treated with vehicle (CTRL) or GCS inhibitor (PDMP) at different doses (50, 75, 100 μM) during 16 hours. (n=2).

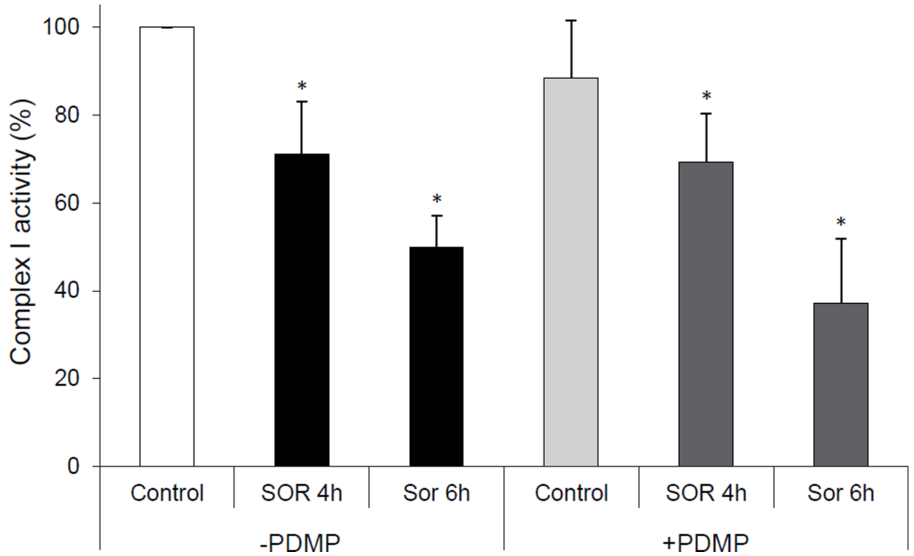

Supplementary Figure S6: Hep3B cells were incubated with sorafenib (10 µM) in the presence of vehicle (-PDMP) or GCS inhibitor (+PDMP) at 50 µM, and complex I activity measured in protein extracts obtained at different times (n=3). *, $p<0.05$ vs. control Hep3B cells.

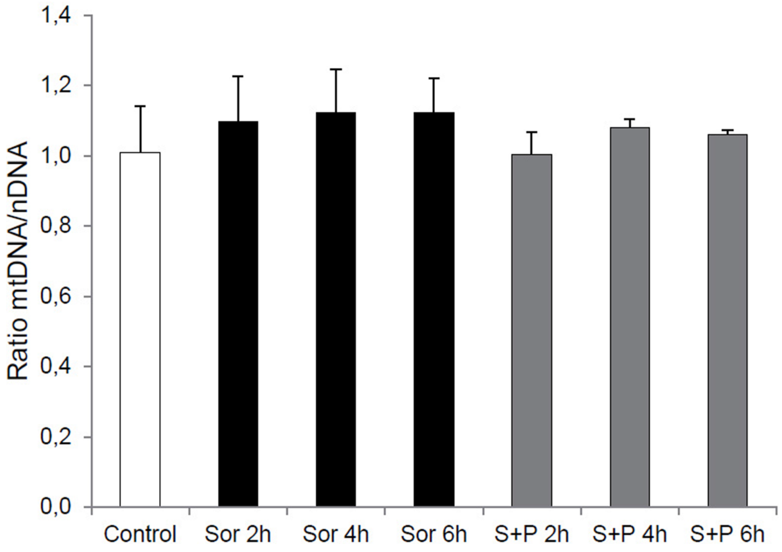

Supplementary Figure S7: Hep3B cells were incubated with sorafenib (10 µM) in the presence of vehicle (Sor) or GCS inhibitor (S+P) and the ratio between the mitochondrial and nuclear DNA was measured in DNA samples obtained at different times (n=3).

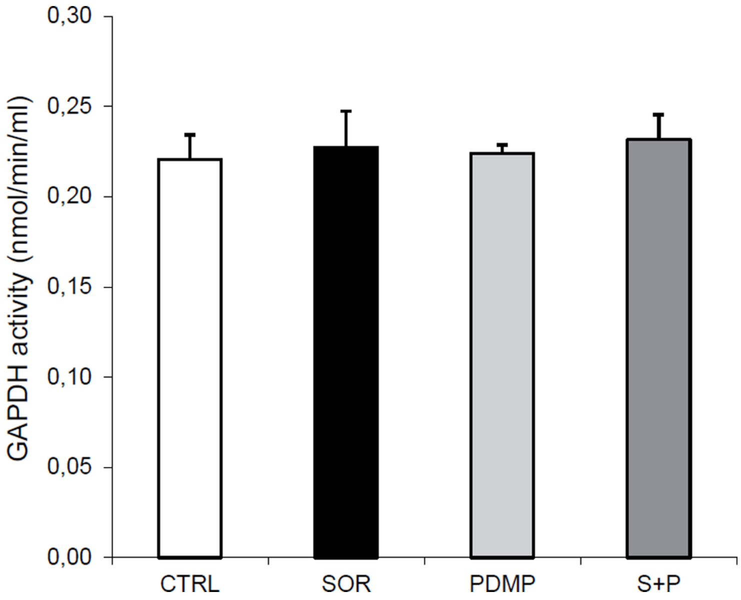

Supplementary Figure S8: GAPDH expression was measured after 6 hours in Hep3B cells treated with sorafenib (10 µM and/or PDMP combination (50 µM). (n=3).

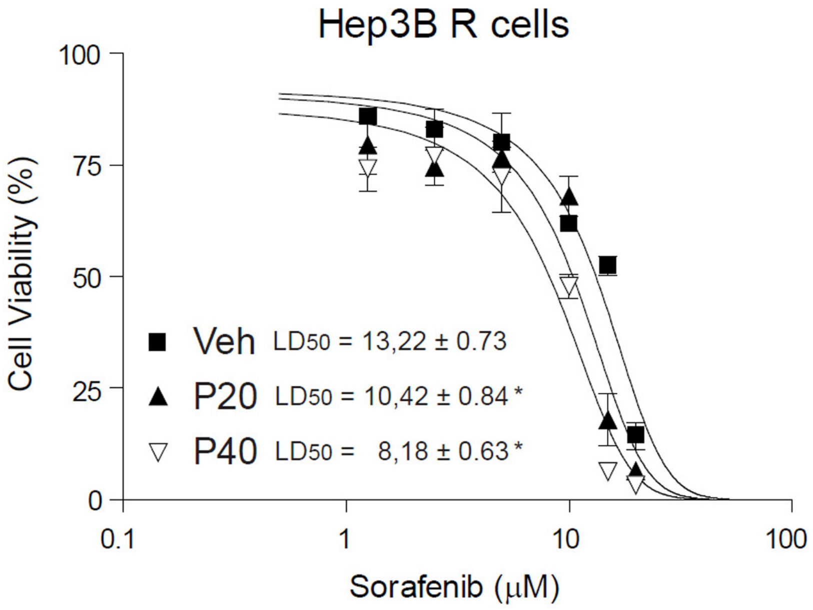

Supplementary Figure S9: Dose response analysis of cell viability after sorafenib administration in sorafenib-resistant Hep3B cells treated with vehicle or GCS inhibitor (PDMP) at 20 and 40 μM. (n=3). *, $p<0.05$ vs. vehicle-treated Hep3B R cells.

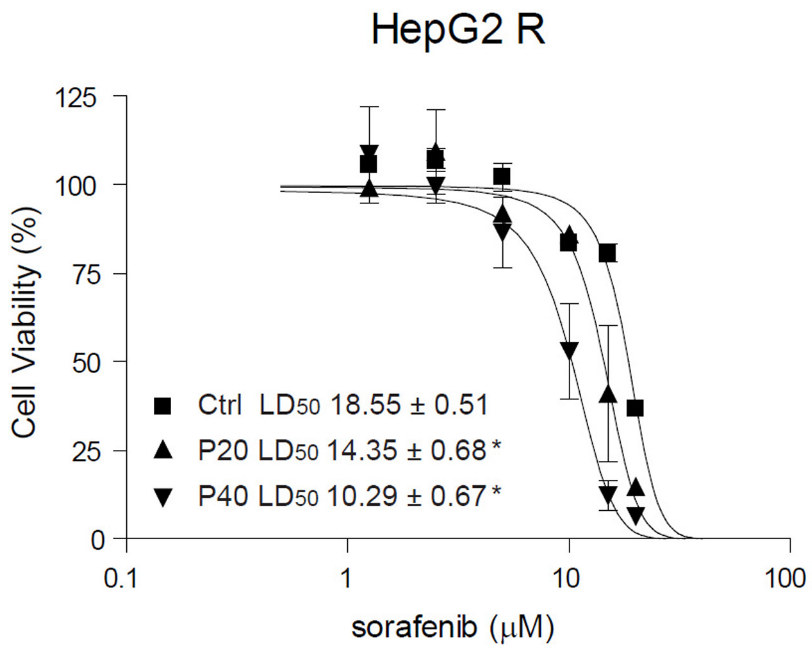

Supplementary Figure S10: Dose response analysis of cell viability after sorafenib administration in sorafenib-resistant HepG2 cells treated with vehicle or GCS inhibitor (PDMP) at 20 and 40 μM. (n=3). *, $p<0.05$ vs. vehicle-treated HepG2 R cells.

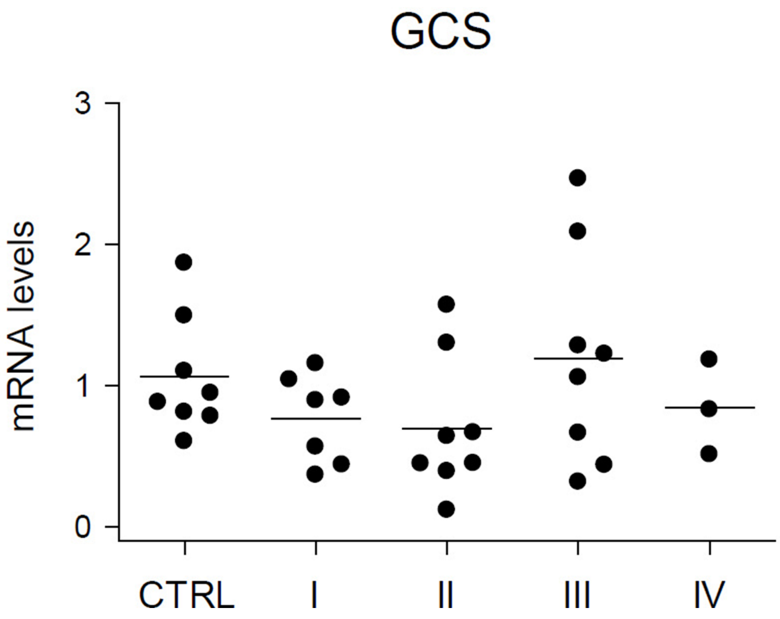

Supplementary Figure S11: GCS mRNA levels measured in a liver cancer cDNA array (Origene) containing cDNA from normal liver or tumoral tissue from HCC patients. This array covers 8 normal, 7 stage I, 8 stage II, 8 stage IIIA and 3 stage IV pathologist-verified tissues. The results are normalized and validated with β-actin.

Supplementary Table S1: mRNA levels of sphingolipidic enzymes in HepG2 cells after sorafenib exposure

Sorafenib (µM)	0	5
ASMase	1.00±0.03	1.68±0.05 *
ACDase	1.00±0.08	0.76±0.16
CerS2	1.00±0.12	0.99±0.03
GCS	1.00±0.07	1.83±0.08 *
SPT	1.00±0.06	1.00±0.06
SK1	1.00±0.03	0.96±0.04

HepG2 cells were exposed to sorafenib (5 µM) for 16 hours and enzymes in ceramide metabolism analyzed by RT-PCR. (n=2). *, $p<0.05$ vs. control.

Supplementary Table S2: mRNA levels of sphingolipidic enzymes in sorafenib-resistant and sensitive HepG2 cells

	HepG2 S	HepG2 R
ASMase	1.00±0.37	0.60±0.22
ACDase	1.00±0.06	1.39±0.43
CerS2	1.00±0.17	1.19±0.12
GCS	1.00±0.07	1.71±0.38 *
SPT	1.00±0.15	0.95±0.15
SK1	1.00±0.16	1.05±0.72

Ceramide-related enzymes were analyzed by RT-PCR in HepG2 cells that exhibit sorafenib resistance after long-term exposure to sorafenib (HepG2 R) or vehicle (HepG2 S). (n=3). *, $p<0.05$ vs. control.
